# SARS-CoV-2 in Captive Nonhuman Primates, Spain, 2020–2023

**DOI:** 10.3201/eid3006.231247

**Published:** 2024-06

**Authors:** David Cano-Terriza, Adrián Beato-Benítez, Leira Fernández-Bastit, Joaquim Segalés, Júlia Vergara-Alert, Eva Martínez-Nevado, Andrea Carretero, Dietmar Crailsheim, Pilar Soriano, Javier Planas, Mario Torro, Ignacio García-Bocanegra

**Affiliations:** Universidad de Córdoba, Córdoba, Spain (D. Cano-Terriza, A. Beato-Benítez, I. García-Bocanegra);; CIBERINFEC ISCIII, Madrid, Spain (D. Cano-Terriza, I. García-Bocanegra);; Universitat Autònoma de Barcelona, Barcelona, Spain (L. Fernández-Bastit, J. Segalés, J. Vergara-Alert);; Zoo Aquarium de Madrid, Madrid (E. Martínez-Nevado);; Centro de Rescate de Primates RAINFER/Fundación Chimpatía, Madrid (A. Carretero);; Fundació Mona, Girona, Spain (D. Crailsheim);; Río Safari Elche, Alicante, Spain (P. Soriano);; Oasis Wildlife Fuerteventura, La Lajita, Canary Islands, Spain (J. Planas);; Terra Natura Benidorm, Benidorm, Spain (M. Torro)

**Keywords:** COVID-19, SARS-CoV-2, nonhuman primates, respiratory infections, severe acute respiratory syndrome coronavirus 2, SARS, coronavirus disease, zoonoses, viruses, coronavirus, Spain

## Abstract

We conducted a serologic and molecular study to assess exposure of captive nonhuman primates (NHPs) to SARS-CoV-2 in Spain during the 2020–2023 COVID-19 pandemic. We found limited exposure of NHPs to SARS-CoV-2. Biosafety measures must be strictly maintained to avoid SARS-CoV-2 reverse-zoonotic transmission in the human–NHP interface.

COVID-19 caused by SARS-CoV-2 is an emerging respiratory disease that likely originated from wildlife in late 2019 (*1*). Although the main driver of SARS-CoV-2 spread is human-to-human transmission, several wild and domestic mammals are susceptible to SARS-CoV-2 infection; natural infections have been reported in 29 nonhuman animal species (*2*).

Nonhuman primates (NHPs) are among the most susceptible taxonomic groups to SARS-CoV-2 infection (*3*) and are suitable animal models to evaluate SARS-CoV-2 pathogenesis (*4*). Natural infections with clinical outcomes have been reported in both captive and free-living NHPs worldwide (*2*,*5*–*8*). Because active surveillance of SARS-CoV-2 has not been conducted in NHPs, we assessed SARS-CoV-2 circulation among NHPs housed in zoos and rescue centers in Spain, where NHPs can be found in proximity to humans.

## The Study

**Figure 1:**
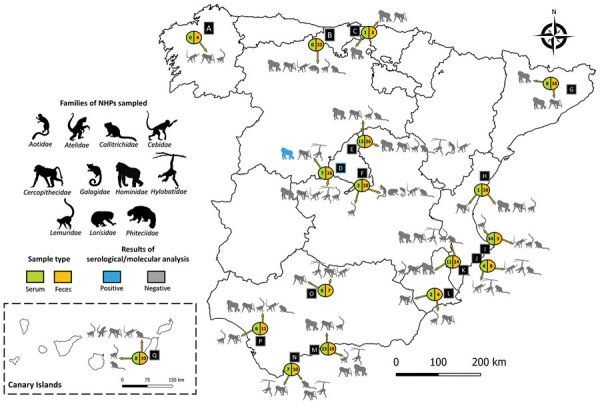
Geographic distribution of captive NHPs sampled in study of SARS-CoV-2 in Spain, 2020–2023. Serum and fecal samples were collected from different NHP species at 17 zoos and NHPs rescue centers (letters A–Q) in Spain during January 2020–March 2023. Inset indicates the specimens collected in the Canary Islands, Spain. Animal images indicate the families of NHPs examined. Blue image indicates the 2 gorillas that were SARS-CoV-2 seropositive. Circles show the total numbers of serum or fecal samples analyzed at each zoo or rescue center. Arrows indicate which NHP families underwent serum or fecal sample testing for SARS-CoV-2. NHP, nonhuman primate.

During January 2020–March 2023, we collected serum samples from 127 different NHPs belonging to 30 species housed in 17 zoos and NHP rescue centers in Spain. The number of sampled animals represented ≈40% of the total census of those species in the selected zoos and rescue centers. We collected 16 serum samples in 2020, 76 in 2021, 28 in 2022, and 7 in 2023 (Table; Figure 1). In addition, we collected 186 fresh fecal samples during February–May 2022 within the same zoos and NHPs rescue centers, which comprised 39 samples collected from animals housed in individual facilities and 147 pooled samples from the floors of facilities with >1 animal (1 pool per facility and species) belonging to 64 different NHP species (Appendix Table). The study did not require additional blood extractions because we obtained all serum samples from serum banks or from NHPs that had medical check-ups or surgical interventions during the study period.

**Table:** Distribution of serum samples in study of SARS-CoV-2 in captive nonhuman primates in Spain, 2020–2023*

NHP family and species	No. positive/total†	Zoos and rescue centers‡																
		A	B	C	D	E	F	G	H	I	J	K	L	M	N	O	P	Q
Callitrichidae																		
* Callithrix geoffroyi*	0/1	0	0	0	0	0	0	0	0	0	0	0	0	0	0	0	0	1
* Callithrix jacchus*	0/3	0	0	0	0	0	0	0	0	0	0	0	0	0	0	0	0	3
* Leonthopithecus rosalia*	0/1	0	0	0	0	0	0	0	0	0	1	0	0	0	0	0	0	0
* Mico argentatus*	0/2	0	0	0	0	0	0	0	0	0	0	0	0	0	0	0	0	2
* Saguinus oedipus*	0/2	0	0	0	0	1	0	0	0	0	0	0	0	0	0	0	0	1
Subtotal	0/9	0	0	0	0	1	0	0	0	0	1	0	0	0	0	0	0	7
Cebidae																		
* Sapajus apella*	0/1	0	0	0	0	0	0	0	0	0	1	0	0	0	0	0	0	0
Subtotal	0/1	0	0	0	0	0	0	0	0	0	1	0	0	0	0	0	0	0
Cercopithecidae																		
* Cercocebus atys lunulatus*	0/6	0	0	0	0	0	0	0	0	0	0	0	0	0	0	4	2	0
* Cercopithecus mitis*	0/1	0	0	0	0	1	0	0	0	0	0	0	0	0	0	0	0	0
* Cercopithecus neglectus*	0/3	0	0	0	0	0	0	0	0	0	0	0	2	0	0	1	0	0
* Colobus guereza*	0/1	0	0	0	0	0	0	0	0	0	0	0	0	0	1	0	0	0
* Lophocebus aterrimus*	0/1	0	0	0	0	0	0	0	0	0	0	1	0	0	0	0	0	0
* Macaca sylvanus*	0/11	0	0	0	0	3	0	0	0	0	0	0	0	0	5	3	0	0
* Macaca tonkeana*	0/1	0	0	0	0	1	0	0	0	0	0	0	0	0	0	0	0	0
* Mandrillus leucophaeus*	0/1	0	0	0	0	0	0	0	1	0	0	0	0	0	0	0	0	0
* Mandrillus sphinx*	0/1	0	0	0	1	0	0	0	0	0	0	0	0	0	0	0	0	0
* Miopithecus ogouensis*	0/2	0	0	0	0	0	0	0	0	0	0	0	0	2	0	0	0	0
* Papio cynocephalus*	0/1	0	0	0	1	0	0	0	0	0	0	0	0	0	0	0	0	0
Subtotal	0/29	0	0	0	2	5	0	0	1	0	0	1	2	2	6	8	2	0
Hominidae																		
* Gorilla gorilla gorilla*	2/3	0	0	1	**2**	0	0	0	0	0	0	0	0	0	0	0	0	0
* Pan troglodytes*	0/4	0	0	0	0	0	0	0	0	0	0	4	0	0	0	0	0	0
* Pan troglodytes ellioti*	0/1	0	0	0	0	0	0	1	0	0	0	0	0	0	0	0	0	0
* Pan troglodytes troglodytes*	0/3	0	0	0	1	0	0	2	0	0	0	0	0	0	0	0	0	0
* Pan troglodytes verus*	0/1	0	0	0	0	0	0	1	0	0	0	0	0	0	0	0	0	0
* Pan troglodytes verus/troglodytes*	0/4	0	0	0	0	0	0	4	0	0	0	0	0	0	0	0	0	0
* Pongo pygmaeus*	0/6	0	0	0	1	1	0	0	0	0	0	2	0	2	0	0	0	0
Subtotal	2/22	0	0	1	4	1	0	8	0	0	0	6	0	2	0	0	0	0
Hylobatidae																		
* Hylobates lar*	0/3	0	0	0	1	0	0	0	0	0	1	0	0	0	1	0	0	0
* Hylobates syndactylus*	0/1	0	0	0	0	0	0	0	0	0	1	0	0	0	0	0	0	0
Subtotal	0/4	0	0	0	1	0	0	0	0	0	2	0	0	0	1	0	0	0
Lemuridae																		
* Eulemur macaco*	0/3	0	0	0	0	0	0	0	0	0	0	0	0	3	0	0	0	0
* Lemur catta*	0/38	0	0	0	0	6	2	0	0	17	0	4	0	5	0	0	4	0
* Varecia rubra*	0/1	0	0	0	0	0	0	0	0	0	0	0	0	1	0	0	0	0
* Varecia variegata*	0/20	0	0	0	0	0	0	0	0	17	0	0	0	2	0	0	0	1
Subtotal	0/62	0	0	0	0	6	2	0	0	34	0	4	0	11	0	0	4	1
Grand total	2/127	0	0	1	7	13	2	8	1	34	4	11	2	15	7	8	6	8

*Sampling was conducted during January 2020–March 2023. Numbers indicate the number of NHP species tested for SARS-Co-V-2 at each zoo or rescue center. Bold number indicates the 2 SARS-CoV-2–seropositive gorillas identified at zoo D. NHP, nonhuman primate. †Number of SARS-CoV-2–seropositive animals per the total number of animals tested at each zoo or rescue center. ‡Serum samples were obtained from zoos or rescue centers represented by letters A–Q. Locations of the zoos and rescue centers are shown in Figure 1.

We tested all serum samples for antibodies against SARS-CoV-2 nucleocapsid (N) and spike (S) proteins by using 2 commercial multispecies ELISAs (ID Screen SARS-CoV-2 Double Antigen and NeutraLISA SARS-CoV-2; Euroimmun, https://www.euroimmun.com) according to the manufacturer’s instructions. We analyzed ELISA-positive serum samples further by using a virus neutralization test (VNT) as previously described (*9*). In brief, we preincubated 100 SARS-CoV-2 (B.1 lineage) 50% tissue culture infectious doses with 2-fold serial dilutions (1:20 to 1:10,240) of heat-inactivated serum samples in Nunc 96-well cell culture plates (Thermo Fisher Scientific, https://www.thermofisher.com) for 30 minutes at 37°C. Then, we added the virus-serum mixtures onto Vero cells (ATCC, https://www.atcc.org) and read results after 72 hours by using the Cell Titer Glo kit (Promega, https://www.promega.com). We normalized values and calculated VNT_50_ (the reciprocal dilution inhibiting 50% of Vero cell infection) by plotting and fitting the log of serum dilution versus normalized response in Prism 8.4.3 (Graphpad, https://www.graphpad.com). We performed VNTs in duplicate for each sample.

We tested fecal samples for SARS-CoV-2 RNA by using quantitative reverse transcription PCR. We extracted RNA from feces by using the IndiSpin QIAcube HT Pathogen Kit (Indical Biosciences, https://www.indical.com) according to the manufacturer’s instructions. We detected SARS-CoV-2 RNA by using a previously published method (*10*) that had minor modifications adapting it to the Applied Biosystems AgPath-ID One-Step RT-PCR Kit (Thermo Fisher Scientific). We performed PCR amplification by using an Applied Biosystems 7500 Fast Real-Time PCR System (Thermo Fisher Scientific) and considered samples with a cycle threshold value of <40 to be positive for SARS-CoV-2 RNA.

**Figure 2:**
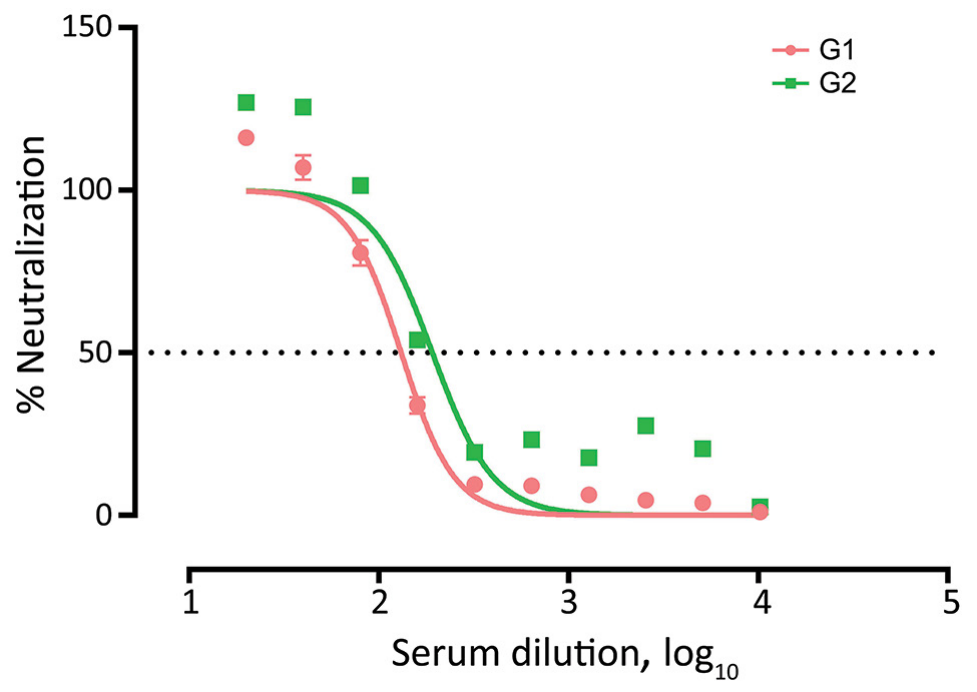
Virus neutralization tests conducted for 2 SARS-CoV-2–seropositive gorillas in study of captive nonhuman primates, Spain, 2020–2023. Serum samples from 2 gorillas tested positive for SARS-CoV-2 antibodies by ELISA. Virus neutralization tests were performed; 50% neutralizing antibody titers were 1:131.4 for G1 and 1:191.9 for G2 serum samples. G1, gorilla 1; G2, gorilla 2.

We confirmed SARS-CoV-2 antibodies in 2/127 (1.6% [95% CI 0.0%–3.7%]) tested animals by both ELISA and VNT. The seropositive animals were 2 western lowland gorillas (*Gorilla gorilla gorilla*) designated as G1 and G2. VNT_50_ titers were 1:131.4 for G1 and 1:191.9 for G2 serum samples (Figure 2).

G1 and G2 were adult female gorillas sampled at zoo D within the same enclosure (Table; Figure 1). G1 arrived at zoo D from a UK zoo on March 4, 2020, after a favorable medical evaluation. During April 7–10, zookeepers reported that G1 was exhibiting a dry cough. A serum sample was collected from G1 on April 24, 2020, during a clinical intervention after an aggression was suffered by another gorilla from the same group. Previous studies of NHPs experimentally infected with SARS-CoV-2 reported clinical signs within the first week after infection (*4*,*11*). Therefore, the exposure of G1 to SARS-CoV-2 most likely occurred during the first wave of the COVID-19 pandemic in Spain (February–June 2020), when the zoo was closed to the public because of lockdown efforts to curb coronavirus cases. G2 was sampled on November 4, 2022, and did not show previous clinical signs compatible with SARS-CoV-2 infection. The 4 group members that shared the same facilities with the 2 seropositive gorillas could not be sampled, but they did not show any indications of disease at that time.

Previous cases of natural SARS-CoV-2 infections have been described in gorillas; the animals experienced asymptomatic or mild illness, involving coughing, congestion, nasal discharge, loss of appetite, and tiredness that did not require medical interventions and resolved within a few days (*5*,*6*,*8*,*12*). In those previous cases, intraspecific transmission among animals from the same facilities was suggested.

Despite the rigorous biosafety protocols used when working with NHPs (e.g., using disinfection mats, masks, gloves, and hand disinfectants and changing overalls), the zoo staff was the most plausible source of transmission to the gorilla troop, which has been suggested for previous outbreaks reported in zoos (*6*,*8*). Nevertheless, exposure of G1 to SARS-CoV-2 at the zoo of origin or during transport, as well as intraspecific transmission, cannot be ruled out.

Fecal shedding of SARS-CoV-2 peaks during the symptomatic period and can persist 1–35 days after onset of clinical signs in humans (*13*,*14*). Similarly, a study of rhesus macaques (*Macaca mulatta*) experimentally infected with SARS-CoV-2 reported that rectal swab samples tested positive for RNA up to 27 days after infection (*15*). In our study, SARS-CoV-2 RNA was not detected in any of the 186 fecal samples tested (Appendix Table), including those obtained at the facility housing G1 and G2. Considering the time of fecal sampling and the virus excretion period in primate species (≈1 month), our findings indicate an absence of virus circulation during this temporal window. Discrepancies between serologic and molecular results in the 2 seropositive gorillas could be explained by differences in antibody persistence, which ranged from 3 to >9 months in experimentally immunized NHPs (Appendix references *16,17*), and by differences in virus RNA excretion in feces, as well as sampling times (April 2020 and November 2022 for serum samples and February 2022 for fecal samples).

The first limitation of this study is that the spatial distribution of sampling was not homogeneous. Approximately 60% of the tested serum samples were from 4 of the sampled centers. Second, serum samples could not be longitudinally analyzed. Because antibodies decay over time (Appendix references *16,17*), SARS-CoV-2 exposure before the sampling period cannot be ruled out. Further longitudinal studies would be of great interest to better understand the temporal dynamics of SARS-CoV-2 in NHPs.

## Conclusions

Our findings indicate a limited SARS-CoV-2 exposure in captive NHPs populations in zoos and NHPs rescue centers in Spain during the 2020–2023 COVID-19 pandemic period. G1 only exhibited mild symptoms, and G2 exhibited no symptoms, highlighting the importance of conducting active screening and surveillance testing to reduce the potential emergence of unidentified reservoirs and virus evolution. Considering the potential health risk and threat to the conservation of NHP species, the possibility of the emergence of new reservoirs, and the opportunities for virus evolution, vaccination of captive animals and the proper use of biosafety measures and personal protective equipment are critical to prevent future reverse-zoonotic transmission of SARS-CoV-2. 

Appendix Additional information for SARS-CoV-2 in captive nonhuman primates, Spain, 2020–2023.
