# Supplementary material for: SARS-CoV-2 in Captive Nonhuman Primates, Spain, 2020–2023
Source: Emerg Infect Dis. 2024 Jun;30(6):1253–7. doi: 10.3201/eid3006.231247 (PMC11139002; doi:10.3201/eid3006.231247)
Supplement: Appendix — Additional information for SARS-CoV-2 in captive nonhuman primates, Spain, 2020–2023. [file 23-1247-Techapp-s1.pdf]

Article DOI: https://doi.org/10.3201/eid3006.231247
EID cannot ensure accessibility for supplementary materials supplied by authors. Readers who have difficulty accessing supplementary content should contact the authors for assistance.

SARS-CoV-2 in Captive Nonhuman Primates, Spain, 2020–2023

Appendix

Appendix Table. Distribution of individual and pooled fecal samples collected from captive NHPs in Spain during February–May 2022*

NHP family and species	No. positive/ total†	Zoos and rescue centers‡																
		A	B	C	D	E	F	G	H	I	J	K	L	M	N	O	P	Q
Aotidae																		
Aotus azarae	0/1	0	0	0	0	1	0	0	0	0	0	0	0	0	0	0	0	0
Aotus nancymae	0/1	0	0	0	0	0	1	0	0	0	0	0	0	0	0	0	0	0
Subtotal	0/2	0	0	0	0	1	1	0	0	0	0	0	0	0	0	0	0	0
Atelidae																		
Ateles geoffroyi	0/1	0	0	0	0	0	0	0	0	0	0	0	0	0	0	0	0	1
Subtotal	0/1	0	0	0	0	0	0	0	0	0	0	0	0	0	0	0	0	1
Callitrichidae																		
Callimico goeldii	0/5	0	2	0	0	0	1	0	0	0	1	0	0	0	1	0	0	0
Callithrix geoffroyi	0/8	0	1	0	0	3	0	0	0	1	0	0	0	0	1	0	1	1
Callithrix jacchus	0/5	0	0	0	0	1	1	0	0	0	1	1	0	0	0	0	0	1
Cebuella pygmaea	0/2	0	0	0	0	0	0	0	0	0	1	0	0	0	0	0	0	1
Leontopithecus chrysomelas	0/3	0	1	0	0	1	0	0	0	0	0	0	0	0	0	0	1	0
Leonthopithecus rosalia	0/2	0	1	0	0	0	1	0	0	0	0	0	0	0	0	0	0	0
Mico argentatus	0/2	0	1	0	0	0	0	0	0	0	0	0	0	0	0	0	0	1
Saguinus bicolor	0/1	0	1	0	0	0	0	0	0	0	0	0	0	0	0	0	0	0
Saguinus imperator	0/2	0	1	0	0	0	0	0	0	0	0	0	0	0	1	0	0	0
Saguinus labiatus	0/2	0	1	0	0	0	0	0	0	0	0	0	0	0	0	0	0	1
Saguinus midas	0/2	0	1	0	0	0	0	0	0	1	0	0	0	0	0	0	0	0
Saguinus oedipus	0/7	0	1	0	0	2	0	0	0	0	1	1	0	0	0	0	1	1
Subtotal	0/41	0	11	0	0	7	3	0	0	2	4	2	0	0	3	0	3	6
Cebidae																		
Cebus olivaceus	0/1	0	0	0	0	0	0	0	0	0	0	0	0	0	0	0	1	0
Saimiri boliviensis	0/1	0	0	0	0	0	0	0	0	0	1	0	0	0	0	0	0	0
Saimiri sciureus	0/5	0	1	0	0	1	1	0	0	1	0	0	0	0	0	1	0	0
Sapajus apella	0/8	1	0	0	2	2	1	0	0	0	1	0	0	0	0	0	0	1
Subtotal	0/15	1	1	0	2	3	2	0	0	1	2	0	0	0	0	1	1	1
Cercopithecidae																		
Cercocebus atys lunulatus	0/3	0	0	0	0	0	0	0	1	0	0	0	0	0	0	1	1	0
Cercocebus chrysogaster	0/1	0	0	0	0	0	0	0	0	0	0	1	0	0	0	0	0	0
Cercopithecus mona	0/1	0	0	0	0	1	0	0	0	0	0	0	0	0	0	0	0	0
Cercopithecus neglectus	0/4	0	1	1	0	0	0	0	0	0	0	0	1	0	0	1	0	0
Chlorocebus aethiops	0/2	0	0	0	0	1	0	0	0	0	0	0	0	0	1	0	0	0
Colobus guereza	0/5	0	1	0	1	0	0	0	0	0	0	0	1	0	2	0	0	0
Erythrocebus patas	0/1	0	0	0	0	0	0	0	0	0	0	0	0	0	0	0	0	1
Lophocebus aterrimus	0/4	0	0	0	0	0	0	0	0	0	0	4	0	0	0	0	0	0
Macaca fascicularis	0/2	1	0	0	0	0	0	0	0	0	0	0	0	0	0	0	1	0
Macaca silenus	0/1	0	1	0	0	0	0	0	0	0	0	0	0	0	0	0	0	0
Macaca sylvanus	0/9	0	0	0	0	1	0	3	0	0	0	0	0	0	3	1	1	0
Macaca tonkeana	0/1	0	0	0	0	1	0	0	0	0	0	0	0	0	0	0	0	0
Mandrillus leucophaeus	0/2	0	0	0	0	0	0	0	1	0	0	0	0	0	0	1	0	0
Mandrillus sphinx	0/2	0	0	0	1	0	0	0	0	0	0	0	0	0	0	0	1	0
Miopithecus ogouensis	0/7	0	0	0	0	2	0	0	2	0	0	0	0	2	0	1	0	0
Papio cynocephalus	0/4	0	0	0	4	0	0	0	0	0	0	0	0	0	0	0	0	0
Papio hamadryas	0/1	1	0	0	0	0	0	0	0	0	0	0	0	0	0	0	0	0
Papio papio	0/1	0	0	1	0	0	0	0	0	0	0	0	0	0	0	0	0	0
Subtotal	0/51	2	3	2	6	6	0	3	4	0	0	5	2	2	6	5	4	1
Galagidae																		

NHP family and species	No. positive/ total†	Zoos and rescue centers‡																
		A	B	C	D	E	F	G	H	I	J	K	L	M	N	O	P	Q
Galago moholi	0/3	0	0	0	0	0	3	0	0	0	0	0	0	0	0	0	0	0
Subtotal	0/3	0	0	0	0	0	3	0	0	0	0	0	0	0	0	0	0	0
Hominidae																		
Gorilla gorilla gorilla	0/4	0	0	1	1	0	0	0	1	0	0	0	0	1	0	0	0	0
Pan troglodytes	0/11	0	1	0	1	3	0	1	1	0	0	3	0	0	0	0	1	0
Pan troglodytes ellioti	0/2	0	0	0	0	0	0	2	0	0	0	0	0	0	0	0	0	0
Pan troglodytes troglodytes	0/4	0	0	0	0	0	0	4	0	0	0	0	0	0	0	0	0	0
Pan troglodytes verus	0/3	0	0	0	0	0	0	2	0	0	0	0	0	1	0	0	0	0
Pan troglodytes verus/troglodytes	0/4	0	0	0	0	0	0	4	0	0	0	0	0	0	0	0	0	0
Pongo abelii	0/2	0	2	0	0	0	0	0	0	0	0	0	0	0	0	0	0	0
Pongo pygmaeus	0/4	0	0	0	1	1	0	0	0	0	0	1	0	1	0	0	0	0
Subtotal	0/34	0	3	1	3	4	0	13	2	0	0	4	0	3	0	0	1	0
Hylobatidae																		
Hylobates agilis	0/1	0	0	0	0	1	0	0	0	0	0	0	0	0	0	0	0	0
Hylobates lar	0/5	0	0	0	1	1	0	0	0	0	1	1	0	0	1	0	0	0
Hylobates muelleri	0/1	0	0	0	1	0	0	0	0	0	0	0	0	0	0	0	0	0
Hylobates syndactylus	0/1	0	0	0	0	0	0	0	0	0	1	0	0	0	0	0	0	0
Nomascus gabriellae	0/1	0	0	0	0	0	0	0	0	0	0	0	0	1	0	0	0	0
Nomascus leucogenys	0/1	0	0	0	0	0	0	0	0	0	0	0	0	0	0	1	0	0
Nomascus siki	0/1	0	0	0	0	1	0	0	0	0	0	0	0	0	0	0	0	0
Subtotal	0/11	0	0	0	2	3	0	0	0	0	2	1	0	1	1	1	0	0
Lemuridae																		
Eulemur albifrons	0/1	1	0	0	0	0	0	0	0	0	0	0	0	0	0	0	0	0
Eulemur fulvus	0/2	0	0	0	1	0	0	0	0	0	0	1	0	0	0	0	0	0
Eulemur macaco	0/3	0	2	0	0	0	0	0	0	0	0	0	0	1	0	0	0	0
Eulemur mongoz	0/1	0	0	0	0	0	0	0	1	0	0	0	0	0	0	0	0	0
Eulemur rubriventer	0/1	0	0	0	0	0	0	0	1	0	0	0	0	0	0	0	0	0
Eulemur rufifrons	0/1	0	0	0	0	0	0	0	1	0	0	0	0	0	0	0	0	0
Lemur catta	0/10	0	1	0	1	1	0	0	1	1	0	1	1	1	0	0	1	1
Varecia rubra	0/1	0	0	0	0	0	0	0	0	0	0	0	0	1	0	0	0	0
Varecia variegata	0/6	0	0	0	1	1	0	0	0	1	0	0	1	1	0	0	1	0
Subtotal	0/26	1	3	0	3	2	0	0	4	2	0	2	2	4	0	0	2	1
Lorisidae																		
Nycticebus pygmaeus	0/1	0	0	0	0	0	1	0	0	0	0	0	0	0	0	0	0	0
Subtotal	0/1	0	0	0	0	0	1	0	0	0	0	0	0	0	0	0	0	0
Pitheciidae																		
Pithecia pithecia	0/1	0	1	0	0	0	0	0	0	0	0	0	0	0	0	0	0	0
Subtotal	0/1	0	1	0	0	0	0	0	0	0	0	0	0	0	0	0	0	0
Grand total	0/186	4	22	3	16	26	10	16	10	5	8	14	4	10	10	7	11	10

*Numbers indicate the number of NHP species tested for SARS-Co-V-2 at each zoo or rescue center. NHP, nonhuman primate.
†Number of SARS-CoV-2–positive animals per the total number of animals tested at each zoo or rescue center.
‡Fecal samples were obtained from zoos or rescue centers represented by letters A–Q. Locations of the zoos and rescue centers are shown in Figure 1, main text.

Appendix References

16. Wu Y, Wu N, Jia X, Wu Y, Zhang X, Liu Y, et al. Long-term immune response to Omicron-specific mRNA vaccination in mice, hamsters, and nonhuman primates. MedComm (2020). 2023;4:e460. PubMed https://doi.org/10.1002/mco2.460

17. Honda-Okubo Y, Li L, André G, Leong KH, Howerth EW, Bebin-Blackwell AG, et al. An Advax-CpG55.2™ adjuvanted recombinant spike protein vaccine protects cynomolgus macaques from a homologous SARS-CoV-2 virus challenge. Vaccine. 2023;41:4710–8. PubMed https://doi.org/10.1016/j.vaccine.2023.06.063
